# Toxicopathological Evaluation of Hydroethanol Extract of *Dianthus basuticus* in Wistar Rats

**DOI:** 10.1155/2015/348519

**Published:** 2015-10-04

**Authors:** Anofi Omotayo Tom Ashafa, Mutiu Idowu Kazeem

**Affiliations:** Phytomedicine and Phytopharmacology Research Group, Department of Plant Sciences, University of the Free State, Qwaqwa Campus, Phuthaditjhaba 9866, South Africa

## Abstract

*Background. Dianthus basuticus* is a commonly used medicinal plant in Basotho traditional medicine for the treatment of diabetes, but there is no report on its safety or toxicity. Therefore, we evaluated the toxicity profile of the hydroethanol whole plant extract of* Dianthus basuticus* in Wistar rats.* Methods.* Acute toxicity test was performed with single oral administration of 100–3200 mg/kg body weight of* D. basuticus* extract to rats and the animals were observed for 14 days for signs of toxicity. The subacute toxicity experiment was conducted by oral administration of graded doses (200, 400, and 800 mg/kg) of* D. basuticus* extract daily for 28 days. Behavioural changes as well as haematological, biochemical, and histological parameters were then evaluated.* Results. *There was no observable sign of toxicity in the acute toxicity test. There were significant decreases (*P* < 0.05) in the feed and water intake as well as total cholesterol and triglycerides of the* D. basuticus* extract-treated rats in subacute toxicity study. There were no treatment related differences in the haematological, biochemical, and histopathological evaluations.* Conclusions.* Administration of hydroethanol extract of* D. basuticus* may be safe at the dosages tested in this study but its continuous usage can cause anorexia.

## 1. Introduction

One of the widespread diseases in the world today that has defied cure is diabetes mellitus. International Diabetes Federation estimated that 382 million people are suffering from this disease and the number is projected to increase to 552 million in 2035 [1]. Despite the multifaceted approach (use of oral antidiabetic drugs, exercise, and lifestyle changes) taken in the management of this disease, diabetics continue to suffer from its complications. Oral hypoglycemic agents are also associated with several side effects ranging from hypoglycemia, weight gain, and chronic tissue damage [2]. This accounts for global increase in the usage of medicinal plants for the management of this disease.

The majority of the people from African descent use herbal remedies in one form or the other to manage health related problems such as diabetes [3]. Many of the users of plant derived medicines do so because of poverty, easy access, low cost, and perceived belief that all medicinal herbs, being natural, are generally safe and free from undesirable side effects while acting as an effective medicine [4]. However, very often, herbs may interact with medications that result in adverse conditions. Despite recent researches into the efficacy of herbal remedies, medicinal plants are still poorly understood due to lack of systemic nomenclature, good quality control and safety, and/or toxicity information on herbs [5]. Therefore, medicinal plants and their bioactive components should be put through thorough safety and toxicity tests.

*Dianthus basuticus* Burtt Davy belongs to the Caryophyllaceae family. It is distributed in the Eastern Cape, Gauteng, KwaZulu-Natal, Mpumalanga, and Free State provinces of South Africa. It is known as Lesotho Dianthus, Lesotho Carnation, Drakensberg Carnation, and grass of the road in English, Lesothose grootblom-wilde angelier in Afrikaans, or Hlokwa-la-tsela in Sesotho [6]. Among the Basotho, the plant is widely used in the management of diabetes, as immune modulator, and in increasing fertility of bulls [7]. It is also used in the treatment of chest pains, mumps, and infections. The antimicrobial and cytotoxicity investigations on this plant revealed that extracts and fractions from* D. basuticus* inhibited a wide range of pathogenic bacteria at very low concentrations while hydroethanol and ethanol extracts were cytotoxic to brine shrimp nauplii [8].

Currently, there is immense reliance of the Basotho tribe on this species as an antidiabetic, yet, there is no information in the literature on the safety/toxicity of this valuable medicinal plant. The present study, therefore, investigated the effect of 28-day oral administration of* D. basuticus* whole plant extract on the biochemical, haematological, and histopathological parameters in Wistar rats.

## 2. Materials and Methods

### 2.1. Plant Collections

The plant material was collected in January 2013 from multiple population in the field around Qwaqwa within the Golden Gate Mountains (28° 28′′ 111′ S and 28° 48′′ 314′ E; altitude 11950 m). The species abundance was taken into consideration and collections were made in such a way that the existence of species was not threatened. Proper identification and authentication were done at the Bews Herbarium of the University of KwaZulu-Natal, Pietermaritzburg Campus, by Dr. C. J. Potgieter. Herbarium voucher with reference number (LamMed/01/2013/Qhb) was already deposited at the UFS-Qwaqwa Campus Herbarium.

### 2.2. Extract Preparation

100 g of the dried powdered material was extracted in hydroethanol (50 : 50), with constant shaking on Labcon platform shaker (Laboratory Consumables, PTY, Durban, South Africa) for 24 h. The extract was centrifuged (Hermle Laboratory Centrifuge, Lasec, South Africa) and later filtered using Whatman number 1 filter paper. The filtrate was concentrated using rotary evaporator under vacuum and later freeze-dried in a lyophilizer (Ilshin Lab. Co., Ltd., Seoul, Korea). The percentage yield of the extract was 18.71%.

### 2.3. Animals

Wistar rats of both sexes were obtained from the Animal House of the University of the Free State, South Africa, and were acclimatized for 1 week. They were housed in polypropylene cages under a 12 h light/dark cycle at 20–25°C and 50–60% relative humidity. All animals had access to standard rat chow (Epol Feeds, Westville, South Africa) and tap water* ad libitum*. Ethical approval for the study was obtained from the Interfaculty Ethics Committee of the University of the Free State, South Africa, with approval number NR 02/2013 and all experiments were performed according to the Guide for the Care and Use of Laboratory Animals [9].

### 2.4. Acute Toxicity Study

Acute oral toxicity was evaluated in rats in accordance with the Organization for Economic Cooperation and Development (OECD) guidelines [10] with slight modification. Twenty (20) female animals weighing 180–200 g were divided into five groups (A–E) consisting of four animals each. Group A served as control and orally received 1 mL distilled water while groups B to E received 1 mL of 100, 400, 1600, and 3200 mg/kg body weight (b/w) of the extract. All animals were observed for clinical signs including mortality and moribundity, immediately after dosing and at 1, 2, 4, 8, and 12 h and then twice daily for 14 days. Abnormal findings were recorded with the time of onset and disappearance. Body weights and food consumption were measured daily. On the 14th day, all animals were sacrificed and organs of interest (lung, liver, heart, kidney, stomach, and intestine) were observed macroscopically. Since there was no death or any physically observed sign of toxicity, 200, 400, and 800 mg/kg b/w of the extract were selected for the subacute toxicity study.

### 2.5. Repeated Dose 28-Day Oral Toxicity Study

The study applied the OECD guidelines on repeated dose 28-day oral toxicity [11]. Forty (40) male Wistar rats were randomized into four groups of ten animals each. Group 1 (control) was orally administered distilled water. Groups 2 to 4 were orally treated with 200, 400, and 800 mg/kg body weight/day of* D. basuticus* hydroalcohol extract, respectively. The treatment continued for 28 days and the administration was done using metal oropharyngeal cannula. Observations were made twice daily for mortality and changes in general appearance or behaviour. The body weights were recorded every week, and the individual dose was adjusted for the body weight to maintain the target dose level for all rats. In addition, detailed clinical examination as well as measurement of food and water consumption was performed weekly.

#### 2.5.1. Collection of Blood Sample and Isolation of Organs

The rats were humanely sacrificed on the 29th day by halothane euthanasia following fasting for 12–16 h. Aliquot of the blood was collected through cardiac puncture into sample bottles containing EDTA (BD Diagnostics, Preanalytical Systems, Midrand, USA) for haematological analysis while the remaining blood was kept in plain bottles from which serum was collected and stored for biochemical analysis. The rats were quickly dissected and the whole liver, kidneys, hearts, spleens, lungs, and the testes were excised, freed of fat, blotted with clean tissue paper, and then weighed. The organs to body weight ratio was determined by comparing the weight of each organ with the final body weight of each rat. Defined samples of the lung, heart, liver, kidney, and testes were placed in 10% neutral buffered formaldehyde for histopathological examination.

#### 2.5.2. Determination of Haematological Parameters

Using Horiba ABX 80 Diagnostics system (ABX Pentra Montpellier, France), the following analyses were carried out on the whole blood: white blood cell (WBC), red blood cell (RBC), haematocrit (HCT), haemoglobin (HGB), mean corpuscular volume (MCV), mean corpuscular haemoglobin (MCH), mean corpuscular haemoglobin concentration (MCHC), red blood cell distribution width (RDW), neutrophils (NE), lymphocytes (LY), monocytes (MO), eosinophils (EO), basophils (BA), and platelet (PLT).

#### 2.5.3. Determination of Liver and Kidney Function Parameters

Serum was analyzed for total bilirubin (T-BIL), conjugated bilirubin (C-BIL), total protein (TP), albumin (ALB), globulin (GLB), alkaline phosphatase (ALP), gamma-glutamyl transferase (GGT), aspartate aminotransferase (AST), alanine aminotransferase (ALT), urea (UR), uric acid (UA), and creatinine (CRE) using a BS-200 automatic biochemistry analyzer (Mindray Co., Ltd.) while sodium (Na), potassium (K), and calcium (Ca) were analyzed using Roche electrolyte analyzer (AVL9181; Roche, Germany).

#### 2.5.4. Determination of Serum Lipid Profile

Total cholesterol (TC), triacylglycerol (TG), high density lipoprotein cholesterol (HDL-C), and low density lipoprotein cholesterol (LDL-C) were evaluated using the methods of Allain et al. [12], Tietz [13], Grove [14], and Bergmenyer [15] as described in the Quimica Clinica Aplicada assay kits.

#### 2.5.5. Histopathological Examination

The organs (lungs, heart, kidney, liver, and testis) were fixed in 10% (v/v) formaldehyde, dehydrated through ascending grades of ethanol (70%, 90%, and 95% v/v), cleaned in xylene, and embedded in paraffin wax [16]. Tissue sections were prepared and stained with hematoxylin and eosin. The photomicrographs were taken at ×400 using the Leitz, DIALUX research microscope.

### 2.6. Statistical Analysis

Statistical analysis was performed using GraphPad Prism 5 statistical package (GraphPad Software, La Jolla, CA 92037, USA). Data were expressed as means of ten replicates ± SEM and were subjected to analysis of variance (ANOVA) followed by Bonferroni post hoc test. Statistical significance was considered at *P* < 0.05.

## 3. Results

### 3.1. Acute Toxicity Study

After 14 days, no death or signs of toxicity were observed in all the groups of rats treated with different doses (100, 400, 1600, and 3200 mg/kg) of the plant extract.

### 3.2. Subacute Toxicity

#### 3.2.1. Clinical Signs and Mortality

There was no mortality attributed with the administration of* D. basuticus* extract during the period of study. One animal died in the 400 mg/kg/day group on d 16 while another one died in the 200 mg/kg body weight group on d 24 of the experiment. The death of these animals may be due to gavage accident as the body weights and food intake of these animals before death did not reduce when compared to other animals in the same group.

#### 3.2.2. Food and Water Intake

**Figure 1:**
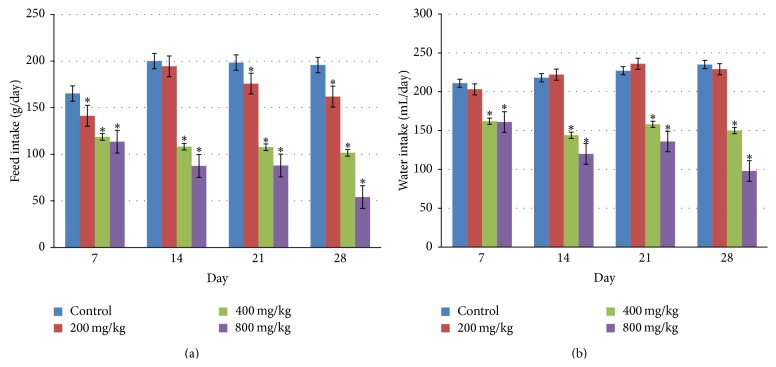
Effect of oral administration of hydroethanol extract of* Dianthus basuticus* on the consumption of (a) feed and (b) water during a 28-day toxicity study in rats. Data are expressed as mean ± SEM,  ^∗^statistical significance at *P* < 0.05.

The weekly mean food and water intake of the rats administered different doses of* D. basuticus* extract is shown in Figure 1. Throughout the duration of the experiment, there were significant reductions (*P* < 0.05) in the food intake between all the extract-treated groups compared to the control. However, only the rats in the 400 and 800 mg/kg groups exhibited significant decrease (*P* < 0.05) in their water intake when compared with control.

#### 3.2.3. Body and Organ Weights

There was no significant difference (*P* > 0.05) between the body weights of the extract-treated groups compared to the control (Table 1). The relative liver weight increased significantly (*P* < 0.001) in the 200 and 800 mg/kg groups while there was significant decrease (*P* < 0.01) in the relative kidney weight of the 400 mg/kg group compared to the control. Significant elevation (*P* < 0.001) was also witnessed in the relative testis weight by the 400 and 800 mg/kg groups while the relative lung weight only rose in the 800 mg/kg group.

#### 3.2.4. Haematological Parameters

Table 2 showed that there was significant decrease (*P* < 0.001) in the MCV as well as increase in the lymphocytes (*P* < 0.05) and platelets (*P* < 0.001) of the animals in the 800 mg/kg group when compared to the control while other haematological parameters were not significantly affected.

#### 3.2.5. Liver and Kidney Functions Parameters

The result of serum liver and kidney function parameters of rats administered hydroethanol extract of* D. basuticus* is presented in Table 3. Animals in the 400 mg/kg group witnessed significant increase (*P* < 0.001) in alkaline phosphatase (ALP) and decrease (*P* < 0.001) in aspartate aminotransferase (AST) compared to the control animals. All other parameters tested were not significantly different in all the groups compared to control.

#### 3.2.6. Lipid Profile

At all the doses tested, there were significant reductions (*P* < 0.001) in the total cholesterol level of the rats compared to the control (Table 4). Animals in the 200 and 400 mg/kg groups witnessed significant increase (*P* < 0.001) in the triglyceride level while it reduces (*P* < 0.001) in the 800 mg/kg group. However, administration of 200 mg/kg of the extract to rats caused significant elevation (*P* < 0.01) in their HDL-C concentration compared to the control.

#### 3.2.7. Histopathological Examination

**Figure 2:**
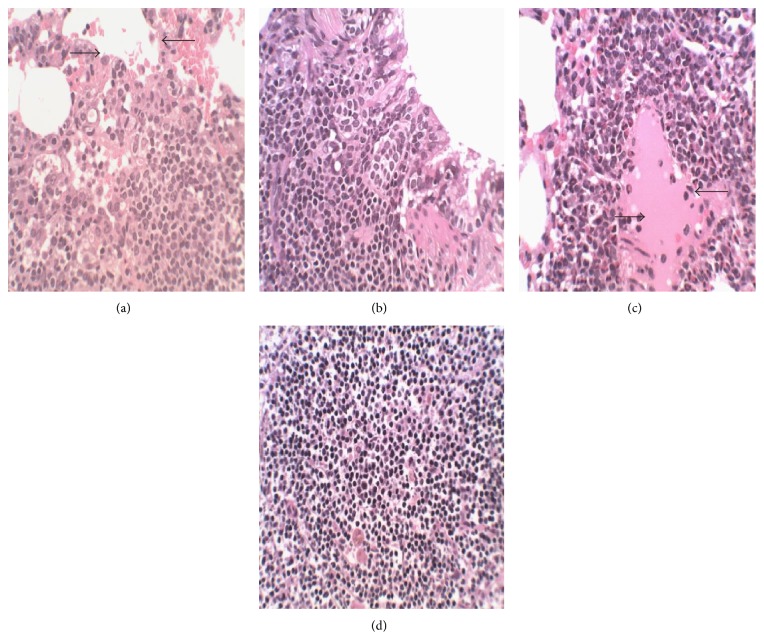
Photomicrograph of section of the lungs of rats following 28-day administration of (a) distilled water, (b) 200 mg/kg, (c) 400 mg/kg, and (d) 800 mg/kg body weight/day of hydroethanol extract of* Dianthus basuticus* (haematoxylin and eosin, ×400).

**Figure 3:**
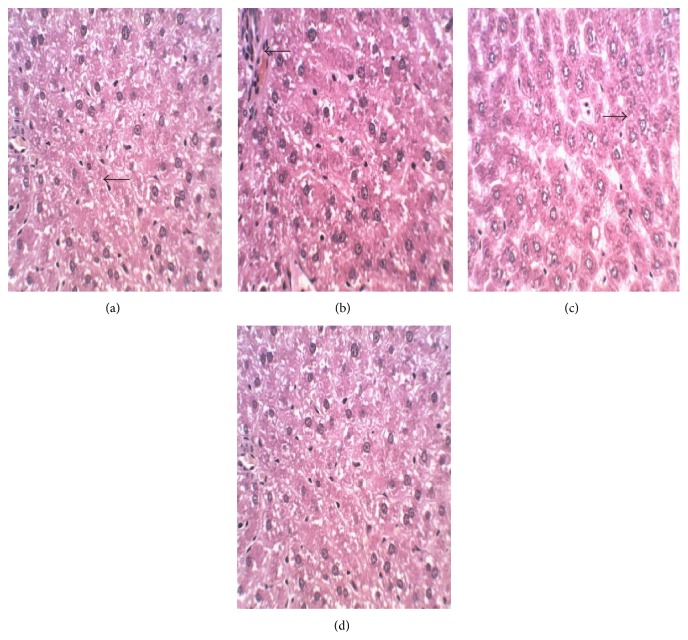
Photomicrograph of section of the liver of rats following 28-day administration of (a) distilled water, (b) 200 mg/kg, (c) 400 mg/kg, and (d) 800 mg/kg body weight/day of hydroethanol extract of* Dianthus basuticus* (haematoxylin and eosin, ×400).

**Figure 4:**
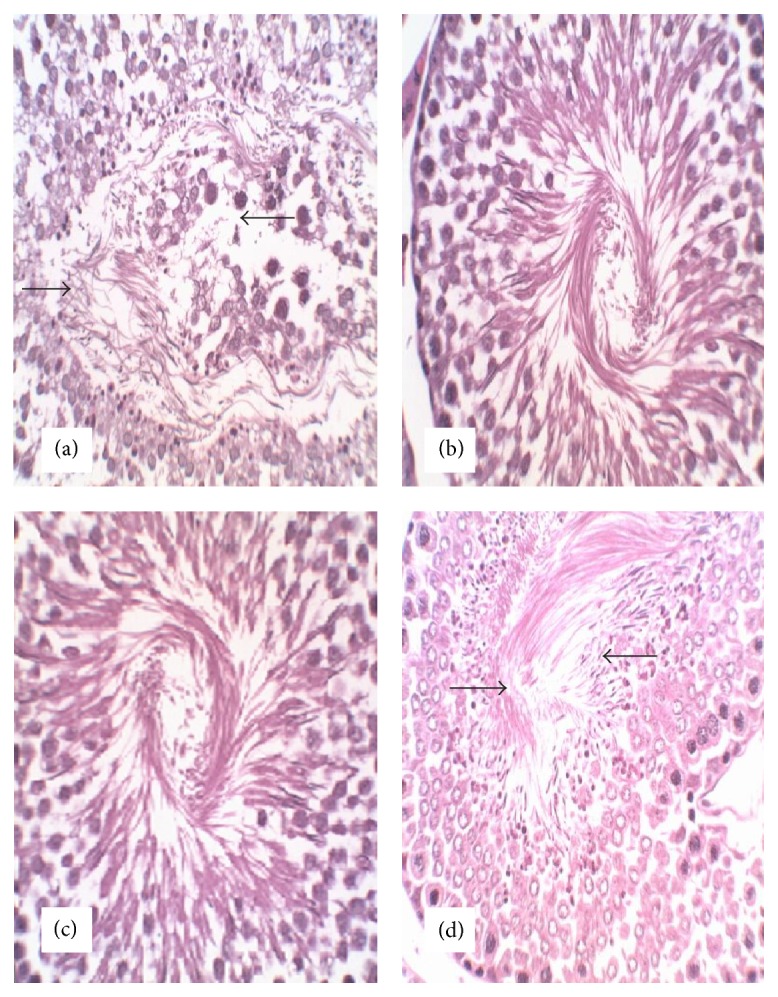
Photomicrograph of section of the testis of rats following 28-day administration of (a) distilled water, (b) 200 mg/kg, (c) 400 mg/kg, and (d) 800 mg/kg body weight/day of hydroethanol extract of* Dianthus basuticus* (haematoxylin and eosin, ×400).

**Figure 5:**
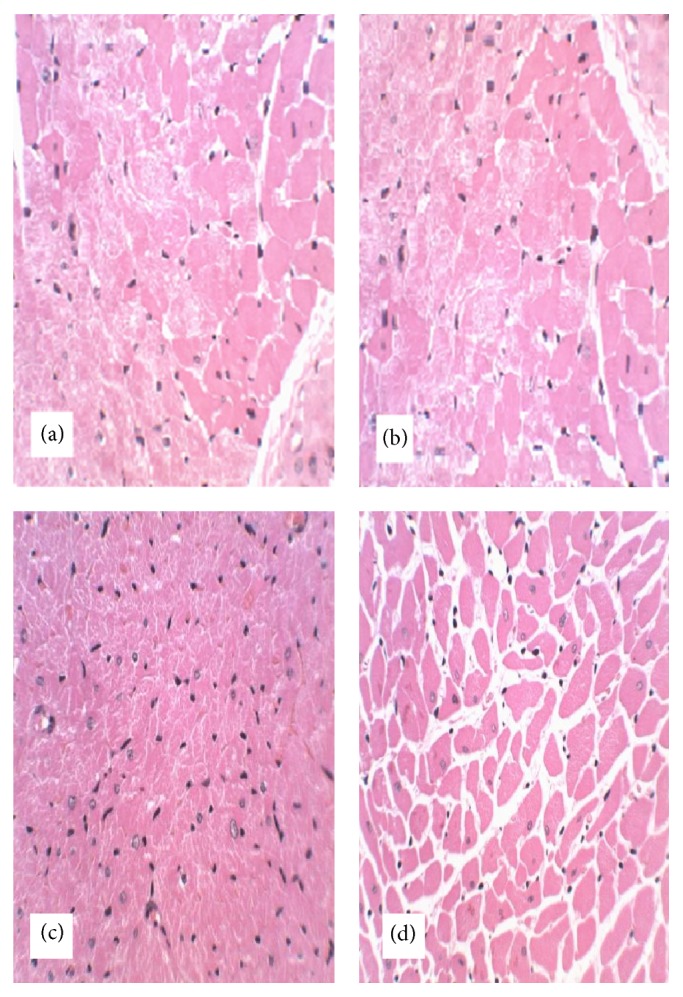
Photomicrograph of section of the heart of rats following 28-day administration of (a) distilled water, (b) 200 mg/kg, (c) 400 mg/kg, and (d) 800 mg/kg body weight/day of hydroethanol extract of* Dianthus basuticus* (haematoxylin and eosin, ×400).

**Figure 6:**
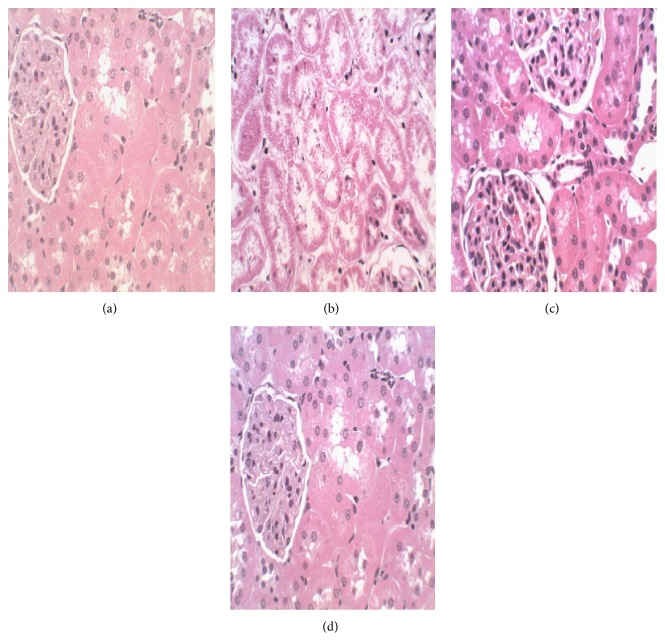
Photomicrograph of section of the kidney of rats following 28-day administration of (a) distilled water, (b) 200 mg/kg, (c) 400 mg/kg, and (d) 800 mg/kg body weight/day of hydroethanol extract of* Dianthus basuticus* (haematoxylin and eosin, ×400).

No gross abnormalities related to the administration of the extract were observed in any of the euthanized animals at the conclusion of the experiment. In the lungs of both control and treated animals, peribronchiolar infiltration of lymphocytes and granulocytes was observed which indicated mild inflammation (Figure 2). There was appearance of swollen glycogen in the liver of the extract-treated rats (Figure 3). Degeneration of testicular tubules, which hitherto indicates sperm formation, was also noted in all the groups tested (Figure 4). However, there were no specific changes visible in the kidneys and hearts of the extract-treated groups compared to the control animals (Figures 5 and 6).

## 4. Discussion

The dose-dependent reduction in food and water consumption of* D. basuticus* extract-treated rats could be an indication that the extract decreases the sense of taste and appetite of the animals. Food and water are essential for life and are required for the growth and development of all organisms. However, the reduction in both the food and water intake did not produce concomitant decrease in the body weight of the animals. Change in the body weights is one of the first critical signs of toxicity [17]. The mean body weights of animals in all experimental groups increased with the duration of the study and were not significantly different from one another. The weight gained by the animals during the experimental period may be an indication that the extract did not hamper the growth of the animals [18]. However, the significant changes in the relative weight of the liver (200 and 800 mg/kg), testis (400 and 800 mg/kg), kidney (400 mg/kg), and lung (800 mg/kg) of the rats may not be regarded as adverse effect because they were not dose-dependent and are not correlated with pathological organ lesions [19].

Haematopoietic system is one of the important parameters used to determine the physiological and pathological status of mammals, as it provides information on the reaction of the body to injury [20]. Though there was significant increase in the platelets counts of the animals in the 800 mg/kg group, this value is still within the physiological range (837–1455 × 10^3^/*μ*L) for Wistar rats [21] and this increase may indicate stimulatory effect on erythropoietin [22]. The observed increase in the lymphocytes level of the 800 mg/kg group may suggest boosting of the immune system of the animals since lymphocytes are the main effector cells of the immune system [22]. However, the reduction in the MCV level may not be regarded as toxic effect because its value in conjunction with that of MCH, MCHC, and RDW relates to the integrity of individual red blood cell [23] while these parameters and red blood cell count were not affected in this study.

Alkaline phosphatase is a marker enzyme of the plasma membrane as well as the endoplasmic reticulum and is present in the cells lining the biliary duct of the liver [24]. Therefore, increase in serum ALP activities may indicate alteration in the permeability of the plasma membrane and cholestatic diseases such as gall stone [25], while alterations in the activity of serum AST may produce consequential effects on the metabolism of amino acids and its biochemical regulation. The significant change in the concentrations of serums ALP and AST in the 400 mg/kg group appears to be biologically irrelevant as there was no dose-response relationship because rats in the highest dose group were not affected. This alteration was not correlated with any pathological lesion in the liver and all other liver function parameters were not affected. It is worthy of note that there was no alteration in all the kidney function parameters (creatinine, urea, uric acid as well as sodium, potassium, and chloride ion) analysed in this study which may be an indication of safety [26].

Alterations in the concentration of lipids like TC, HDL-C, and triglycerides can provide information on the status of lipid metabolism as well as predisposition of the animals to atherosclerosis [27]. The reduction in TC level of animals in all the treated groups may be associated with impairment in the *β*-oxidation of fatty acids [28]. Elevation in triglyceride levels in the 200 and 400 mg/kg groups and its decrease in the 800 mg/kg group may be an indication of addition and depletion of the energy store, respectively, in the animals [29]. However, this may not be toxicologically significant due to the inconsistency in the trend among the dosages. HDL-C is an antiatherogenic factor which is important in the transport of cholesterol from cells to the liver where it is catabolized [30]. Increase in the HDL-C level of all the treated groups which is significant only in the 200 mg/kg group may suggest that there was continuous export of excess cholesterol to the liver for excretion into the bile, thereby reducing the risk of atherosclerosis or coronary artery diseases [31]. This result implies that this extract may be useful in the management of heart related diseases.

There were no treatment related microscopic changes in all the organs observed in this study. The peribronchiolar infiltration of lymphocytes found in the lungs of these animals may possibly be due to inadequate air inspired by these animals since it is found in both the treated and control groups [32]. All morphological changes observed in the liver and testis were randomly distributed between extract-treated and control animals, and the incidences were within the range of normal background lesions. It can therefore be inferred that all histological changes observed were mild and are not considered to be indication of toxicity of the extract.

## 5. Conclusions

These results demonstrate that hydroethanol extract of* Dianthus basuticus* can cause alterations in food and water consumption but did not produce any consistent change in haematological, biochemical, and histopathological parameters of rats. It can therefore be concluded that the administration of this extract at the dosages studied (200–800 mg/kg body weight) may be safe but caution should be taken in its long-term usage as it could lead to anorexia.

## Figures and Tables

**Table 1:** Effect of administration of hydroethanol extract of *Dianthus basuticus* on some weight parameters of male Wistar rats.

Parameters	Dose (mg/kg body weight/day)			
	Control	200	400	800
Initial body weight (g)	257.10 ± 6.90	256.75 ± 3.98	240.81 ± 9.97	275.66 ± 4.22
Final body weight (g)	329.43 ± 6.22	328.66 ± 5.52	309.04 ± 2.94	317.85 ± 6.24
Relative liver weight (%)	3.20 ± 0.01	3.44 ± 0.02^***^	3.21 ± 0.02	3.03 ± 0.01^***^
Relative kidney weight (%)	0.63 ± 0.04	0.64 ± 0.02	0.58 ± 0.03^**^	0.59 ± 0.02
Relative lung weight (%)	0.87 ± 0.04	0.85 ± 0.04	0.89 ± 0.04	1.00 ± 0.05^***^
Relative spleen weight (%)	0.21 ± 0.02	0.19 ± 0.01	0.19 ± 0.01	0.19 ± 0.02
Relative heart weight (%)	0.28 ± 0.02	0.31 ± 0.02	0.30 ± 0.03	0.31 ± 0.01
Relative testis weight (%)	1.07 ± 0.06	1.11 ± 0.03	1.22 ± 0.05^***^	1.15 ± 0.08^***^

Values are presented as mean ± SEM (*n* = 10).

^**^Statistically different from the control at *P* < 0.01.

^***^Statistically different from the control at *P* < 0.001.

**Table 2:** Effect of administration of hydroethanol extract of *Dianthus basuticus* on some haematological parameters of male Wistar rats.

Parameters	Dose (mg/kg body weight/day)			
	Control	200	400	800
White blood cell (×10^3^/*µ*L)	7.77 ± 1.83	8.00 ± 0.98	6.87 ± 2.23	8.55 ± 2.47
Red blood cell (×10^6^/*µ*L)	8.45 ± 0.23	8.91 ± 0.41	8.69 ± 0.31	8.84 ± 0.54
Haematocrit (%)	0.46 ± 0.01	0.46 ± 0.02	0.47 ± 0.02	0.47 ± 0.02
Haemoglobin (g/dL)	15.83 ± 0.15	15.93 ± 0.64	16.10 ± 0.70	16.35 ± 0.64
MCV (fL)	54.67 ± 0.58	54.00 ± 0.00	54.33 ± 1.15	52.50 ± 0.71^***^
MCH (pg)	18.67 ± 0.58	18.00 ± 0.00	18.67 ± 0.58	18.30 ± 0.42
MCHC (g/dL)	34.67 ± 0.57	34.00 ± 0.00	34.00 ± 1.00	35.00 ± 0.0
RDW (fL)	19.20 ± 1.11	19.37 ± 0.49	19.77 ± 1.00	19.70 ± 1.56
Neutrophils (%)	1.76 ± 0.47	1.72 ± 0.65	1.69 ± 0.68	1.32 ± 0.25
Lymphocytes (%)	5.36 ± 1.36	5.72 ± 0.33	5.26 ± 1.47	6.55 ± 1.96^*^
Monocytes (%)	0.32 ± 0.15	0.46 ± 0.11	0.37 ± 0.11	0.36 ± 0.08
Eosinophils (%)	0.13 ± 0.04	0.11 ± 0.07	0.16 ± 0.04	0.31 ± 0.13
Basophils (%)	0.01 ± 0.00	0.01 ± 0.00	0.01 ± 0.01	0.02 ± 0.00
Platelets (×10^3^/*µ*L)	914.67 ± 76.56	881.33 ± 70.63	872.00 ± 59.92	1006.50 ± 43.13^***^

Values are presented as mean ± SEM (*n* = 10).

^*^Statistically different from the control at *P* < 0.05.

^***^Statistically different from the control at *P* < 0.001.

**Table 3:** Liver and kidney function parameters of male Wistar rats administered with hydroethanol extract of *Dianthus basuticus*.

Parameters	Dose (mg/kg body weight/day)			
	Control	200	400	800
Total bilirubin (*µ*mol/L)	5.00 ± 1.00	5.67 ± 2.08	4.00 ± 1.73	7.00 ± 1.41
Conjugated bilirubin (*µ*mol/L)	2.33 ± 0.58	3.00 ± 0.00	1.67 ± 0.58	3.50 ± 0.71
Total protein (g/L)	62.33 ± 4.16	64.00 ± 4.36	65.67 ± 0.68	65.33 ± 0.58
Albumin (g/L)	33.00 ± 0.50	33.33 ± 0.45	33.33 ± 0.06	35.00 ± 0.28
Globulin (g/L)	29.33 ± 4.16	30.67 ± 3.21	32.33 ± 0.57	34.00 ± 0.72
Alkaline phosphatase (U/L)	277.67 ± 26.91	287.67 ± 21.72	314.00 ± 20.48^***^	276.00 ± 4.24
*γ*-Glutamyl transferase (U/L)	3.00 ± 3.46	3.00 ± 2.00	1.33 ± 0.57	4.00 ± 1.41
Aspartate aminotransferase (U/L)	179.00 ± 23.07	168.33 ± 4.04	145.67 ± 13.57^***^	187.50 ± 6.36
Alanine aminotransferase (U/L)	81.67 ± 7.09	75.33 ± 6.43	75.00 ± 5.29	84.00 ± 5.66
Sodium (mmol/L)	144.00 ± 1.00	145.67 ± 1.53	145.67 ± 1.53	142.50 ± 2.12
Potassium (mmol/L)	5.10 ± 0.20	4.93 ± 0.45	4.53 ± 0.31	5.40 ± 0.14
Calcium (mmol/L)	2.48 ± 0.01	2.49 ± 0.06	2.54 ± 0.09	2.46 ± 0.01
Creatinine (mmol/L)	43.00 ± 2.00	36.00 ± 1.53	46.00 ± 5.00	43.00 ± 5.66
Chloride (mmol/L)	103.00 ± 1.00	105.67 ± 1.15	104.33 ± 1.52	105.50 ± 2.12
Urea (mmol/L)	5.90 ± 0.44	7.20 ± 1.08	5.83 ± 0.61	5.60 ± 0.71
Uric acid (mmol/L)	0.09 ± 0.00	0.07 ± 0.01	0.07 ± 0.01	0.10 ± 0.00

Values are presented as mean ± SEM (*n* = 10).

^***^Statistically different from the control at *P* < 0.001.

**Table 4:** Serum lipid profile of male Wistar rats administered with hydroethanol extract of *Dianthus basuticus*.

Parameters	Dose (mg/kg body weight/day)			
	Control	200	400	800
Total cholesterol (mmol/L)	1.46 ± 0.17	1.20 ± 0.07^***^	1.37 ± 0.15^*^	1.20 ± 0.04^***^
Triglycerides (mmol/L)	1.41 ± 0.13	1.60 ± 0.09^***^	1.62 ± 0.12^***^	0.88 ± 0.01^***^
HDL-C (mmol/L)	0.96 ± 0.03	1.07 ± 0.07^**^	0.99 ± 0.06	1.03 ± 0.06
LDL-C (mmol/L)	0.27 ± 0.06	0.27 ± 0.03	0.30 ± 0.01	0.20 ± 0.00
Atherogenic index	0.25 ± 0.01	0.27 ± 0.01	0.28 ± 0.01	0.22 ± 0.01

Values are presented as mean ± SEM (*n* = 10).

^*^Statistically different from the control at *P* < 0.05.

^**^Statistically different from the control at *P* < 0.01.

^***^Statistically different from the control at *P* < 0.001.
